# Dapagliflozin's Association With Cardiorenal Outcomes and Apolipoprotein M Levels in HFrEF Patients: Insights From DEFINE-HF

**DOI:** 10.1016/j.jacadv.2025.101800

**Published:** 2025-06-25

**Authors:** Andrew J. Sauer, Joycie Chang, Zhuxuan Fu, Carla Valenzuela Ripoll, Yoonje Cho, Zhen Guo, Philip Jones, Senthil Selvaraj, Sheryl L. Windsor, Mansoor Husain, Silvio E. Inzucchi, Darren K. McGuire, Bertram Pitt, Benjamin M. Scirica, Bethany A. Austin, Guillermo Umpierrez, Sinh Tran, Björn Dahlbäck, Ali Javaheri, Mikhail N. Kosiborod, DEFINE-HF Investigators

**Affiliations:** a Saint Luke's Mid America Heart Institute, Kansas City, Missouri, USA; b University of Missouri-Kansas City School of Medicine, Kansas City, Missouri, USA; c Washington University in St. Louis, St. Louis, Missouri, USA; d Duke University Medical Center, Duke Molecular Physiology Institute Durham, North Carolina, USA; e Ted Rogers Centre for Heart Research, University of Toronto, Toronto, Ontario, Canada; f Yale University School of Medicine, New Haven, Connecticut, USA; g University of Texas Southwestern Medical Center and Parkland Health and Hospital System, Dallas, Texas, USA; h University of Michigan School of Medicine, Ann Arbor, Michigan, USA; i Cardiovascular Division, Brigham and Women's Hospital and Harvard Medical School, Boston, Massachusetts, USA; j Emory University, Atlanta, Georgia, USA; k Lund University, University Hospital SUS, Malmo, Sweden; l John Cochran VA, St. Louis, Missouri, USA

**Keywords:** apolipoprotein M, cardiorenal effects, dapagliflozin, heart failure with reduced ejection fraction, N-terminal pro B-type natriuretic peptide, urine albumin-creatinine ratio

## Abstract

**Background:**

Apolipoprotein M (ApoM) is associated with lower mortality in heart failure (HF) patients and protects against cardiac and kidney injury in mice.

**Objectives:**

The authors investigated dapagliflozin's cardiorenal effects by studying its association with ApoM in patients with HF with reduced ejection fraction.

**Methods:**

We performed a secondary analysis of DEFINE-HF (Dapagliflozin Effects on Biomarkers, Symptoms, and Functional Status in Patients with HF with Reduced Ejection Fraction) to assess dapagliflozin's effects on ApoM, N-terminal pro B-type natriuretic peptide (NT-proBNP), and urine albumin-creatinine ratio (UACR) changes from baseline to 12 weeks.

**Results:**

Of 263 randomized patients, 236 had ApoM values at baseline (mean 0.641 ± 0.181 μM) and 12 weeks. Dapagliflozin did not significantly affect ApoM vs placebo. However, each 0.1 μM increase in ApoM was associated with a significant decrease in log-transformed NT-proBNP overall (β = −0.11, *P* = 0.006), particularly in dapagliflozin-treated patients (β = −0.19, *P* < 0.001; *P* interaction = 0.025). The inverse relationship between ApoM and NT-proBNP varied by changes in UACR. Dapagliflozin-treated patients with reduced UACR at 12 weeks (n = 53, 22%) experienced a mean NT-proBNP reduction of −0.28 per 0.1 μM increase in ApoM (*P* < 0.001), compared to a smaller reduction in those without UACR change (−0.07, *P* = 0.47). Placebo-treated patients with reduced UACR over 12 weeks did not show significant NT-proBNP changes (β = −0.17, *P* = 0.11).

**Conclusions:**

Dapagliflozin did not significantly alter ApoM overall; however, an inverse association between ApoM and NT-proBNP was observed in dapagliflozin-treated patients with albuminuria. While some NT-proBNP reductions were seen in the placebo group, the significant interaction with treatment allocation suggests a potential dapagliflozin-mediated effect.

Perspectives **COMPETENCY IN MEDICAL KNOWLEDGE:** Using a planned secondary analysis of a randomized trial of patients with HFrEF, we demonstrate that a decrease in NT-proBNP was associated with an increase in the levels of ApoM, particularly in patients assigned to dapagliflozin. The inverse association between change in ApoM and NT-proBNP was particularly notable in patients with increased baseline albuminuria.

**TRANSLATIONAL OUTLOOK:** These findings suggest that subgroups of patients with HF with reduced ejection fraction and albuminuria may have unique ApoM-related responses to dapagliflozin.

Apolipoprotein M (ApoM) is a lipocalin physically bound to high-density lipoprotein^1^ with purported pleiotropic cardioprotective properties.^2^^,^^3^ In human heart failure (HF) studies, low circulating ApoM levels are associated with increased mortality.^2^ Low ApoM levels also partially mediate the association between diabetes and increased risk of cardiovascular death, aborted cardiac arrest, and HF hospitalization.^4^ ApoM binds to sphingosine-1-phosphate (S1P) to activate G protein-coupled receptors, leading to vasoprotective effects such as endothelial cell chemotaxis, wound healing, and angiogenesis.^5^^,^^6^ Additionally, ApoM attenuates inflammation by down-regulating cytokines and enhancing the antioxidative properties of high-density lipoprotein.^7^ ApoM also promotes autophagy in the myocardium.^8^

ApoM may also be protective in the kidney, primarily through the S1P signaling axis. Animal studies have shown that overexpression of ApoM via adenoviral gene transfer of ApoM in hyper-IgA mice suppressed proteinuria and ameliorated the phenotypes of IgA nephropathy. Meanwhile, knockdown of ApoM resulted in increased proteinuria and the expression of fibrosis-related genes such as connective tissue growth factor and collagen-4.^9^ In humans, patients with stage 3 or higher chronic kidney disease (CKD) and cardiovascular disease have lower ApoM levels than those with stage 1 and 2 CKD without cardiovascular disease.^10^

Sodium-glucose cotransporter-2 inhibitors (SGLT2i) improve outcomes for patients with HF through incompletely elucidated mechanisms, but emerging animal studies suggest an interaction with cardioprotective lipoproteins, including ApoM.^11^ SGLT2i induces cellular starvation metabolism, causing lipoprotein levels to increase and energy metabolism to shift toward lipolysis and ketone metabolism.^12^^,^^13^ In HF, the heart relies more heavily on ketone metabolism.^14^ The induction of a fasting state has also been hypothesized to improve endothelial function through the hypoxia-inducible factor-1 pathway. In animal models, the SGLT2i dapagliflozin has been shown to increase ApoM levels and reduce inflammation by attenuating vascular leak and neutrophil recruitment.^11^

In the DEFINE-HF (Dapagliflozin Effects on Biomarkers, Symptoms, and Functional Status in Patients with HF with Reduced Ejection Fraction) study of dapagliflozin vs placebo in patients with HF with reduced ejection fraction (HFrEF), patients treated with dapagliflozin experienced significant improvements in their quality of life compared to the placebo group.^15^ However, the mechanism for the effects on quality of life remains unclear.

Given ApoM's protective effects in the kidneys and heart and dapagliflozin's ability to increase ApoM levels observed in animal models, we analyzed the relationship between dapagliflozin and ApoM to understand how it improves the quality of life in patients with HFrEF. We performed a post hoc secondary analysis in DEFINE-HF to: 1) describe the distribution and correlation of ApoM levels at baseline in the DEFINE-HF population; 2) understand the effect of dapagliflozin on levels of ApoM; and 3) assess the relationship between changes in ApoM and markers of kidney disease and HF. The goal was to provide insights into potential mechanisms by which SGLT2i might mediate protective changes in ApoM and other clinically significant biomarkers such as natriuretic peptides and albuminuria.

## Methods

### Study design

DEFINE-HF was designed primarily to evaluate the effects of dapagliflozin vs placebo on the quality of life in patients with HFrEF 12 weeks post-treatment.^15^ The primary endpoints evaluated in DEFINE-HF included N-terminal pro B-type natriuretic peptide (NT-proBNP), BNP, and scores obtained from the Kansas City Cardiomyopathy Questionnaire (KCCQ). Additional measures, including ApoM levels and urine albumin-creatinine ratios (UACR), were acquired. Data were collected at baseline, 6 weeks, and 12 weeks. Planned secondary analyses included an evaluation of critical biomarkers obtained during the study. Patients included in the study were adult ambulatory patients with or without type 2 diabetes mellitus (T2DM), established HF for at least 16 weeks, left ventricular ejection fraction ≤40%, NYHA functional class II-III HF, and elevated natriuretic peptides. Institutional Review Boards approved the study for all sites, and all patients provided informed consent for research participation.

### Patient selection

Notable exclusion criteria were active HF decompensation, history of type 1 diabetes mellitus, estimated glomerular filtration rate (eGFR) <30 mL/min/1.73 m^2^, recent acute coronary syndrome, percutaneous coronary intervention or cardiac surgery, planned cardiovascular procedural intervention, and history of dapagliflozin sensitivity.

## Biomarkers

Levels of NT-proBNP and all other study laboratory assessments except ApoM were analyzed at a Quest Diagnostics central laboratory, blinded to treatment assignment (NT-ProBNP assay Roche electrochemiluminescent method on Elecsys© platform, Pro-BNP II reagent by Roche/Cobas; BNP assay chemiluminescent method on Siemens ADVIA Centaur platform).

ApoM levels were assessed using plasma samples collected at baseline and 12 weeks. ApoM samples were prepared in Dr Javaheri's lab at Washington University in St. Louis (St. Louis, Missouri, USA) and analyzed with an ELISA at Dr Dahlbӓck's lab at the University of Lund (Lund, Sweden).

### Statistical analysis

Patient demographics, clinical characteristics, medical histories, and labs were described overall and stratified by treatment groups and baseline ApoM tertiles (<0.544, 0.544-0.716, ≥0.716 μM). Continuous measures were summarized by mean ± SD or median (IQR) and compared using 1-way analysis of variance or Kruskal-Wallis rank sum tests, respectively. Categorical variables were reported as counts and percentages and compared using chi-square or Fisher exact tests. A gamma distribution with a log link function was used to account for the skewed distribution of NT-proBNP.

To ensure a robust analysis while minimizing bias due to missing data, we employed a structured approach to handle missing outcomes. If data for an outcome measure were missing at the 12-week visit, we used the value from the next available visit. If no subsequent visit data were available, we imputed the most recent prior visit's value. If no data were available at either time point, the participant was excluded from that specific outcome analysis.

Analysis of ApoM levels at 12 weeks was performed on the modified intention-to-treat data set. Mean ± SD values were reported for ApoM levels at baseline, and paired *t*-tests were used to compare changes from baseline to 12 weeks between treatment groups. To assess the relationship between changes in ApoM levels and key outcomes (log NT-proBNP, 6-minute walk test, weight, and KCCQ scores), we initially used a linear regression model adjusting for baseline values of each respective outcome, baseline ApoM/S1P levels, age, race/ethnicity, sex, eGFR, and T2DM. Treatment groups and the interaction between changes in ApoM and treatment assignment were also included. Stratified analyses by treatment groups were conducted when the interaction term was statistically significant. We also fit a generalized linear model (GLM) with the difference in ApoM from baseline to 12 weeks as the outcome to evaluate the effect of dapagliflozin vs placebo, adjusting for the same covariates.

Given the emerging evidence suggesting that albuminuria may modify the cardiorenal benefits of SGLT2iand recognizing the role of UACR as a biomarker of endothelial dysfunction and microvascular injury, we conducted an analysis to explore its influence on the relationship between ApoM and NT-proBNP. Specifically, we evaluated changes in ApoM stratified by median UACR changes and log-transformed NT-proBNP levels. The median (Q1, Q3) UACR was reported for each subgroup, and linear regression models were conducted, adjusting for baseline ApoM, age, race/ethnicity, sex, eGFR, T2DM, and treatment allocation. All statistical tests were two-tailed and were evaluated at a significance level of 0.05. All analyses were completed using SAS software version 9.4 (SAS Institute Inc) and R software version 4.1.3 (FreeSoftwareFoundation).

## Results

From the overall study population of 263 patients, 131 patients were randomized to dapagliflozin and 132 to placebo. The ApoM levels at baseline (mean 0.641 ± 0.181 μM) and 12 weeks were available for 236 (89.7%) patients. Table 1 describes the characteristics of patients by the tertiles of baseline ApoM levels. Patients with a low baseline ApoM level were more likely to be White than patients with medium and high ApoM levels at baseline (n = 48 vs 49 and 36, 61% vs 62% and 47%, respectively). Patients in each ApoM tertile had similar distributions regarding T2DM, atrial fibrillation, renal function as assessed by eGFR, body mass index, the severity of HF symptoms (as assessed by NYHA class), and natriuretic peptide levels. Compared to patients with medium and high ApoM levels, patients with low baseline ApoM levels were more likely to be male and use lipid-lowering agents and sulfonylureas. Also, patients with low baseline ApoM levels scored lower on the KCCQ overall summary score, clinical summary score, physical limitation score, and social limitation score. Patients excluded from the analysis due to incomplete data are summarized and compared to the larger cohort in Supplemental Table 1. Those with incomplete data were younger (55 vs 62 years), less likely to be taking hydralazine (0% vs 19%), more likely to exhibit NYHA class III symptoms (56% vs 32%), and scored lower on all domains of the KCCQ.

**Table 1:** Characteristics at Baseline Stratified by Baseline ApoM Levels

	Low (<0.544 uM) (n = 79)	Medium (0.544-0.716 uM) (n = 79)	High (≥0.716 uM) (n = 78)	*P* Value
Treatment group				0.85
Dapagliflozin	40 (50.63%)	39 (49.37%)	42 (53.85%)	
Placebo	39 (49.37%)	40 (50.63%)	36 (46.15%)	
Demographics				
Age, y∗	65.18 (9.69)	62.04 (11.96)	58.65 (10.53)	<0.001
Male∗	67 (84.81%)	57 (72.15%)	53 (67.95%)	0.039
Race				0.065
White	48 (60.76%)	49 (62.03%)	36 (46.15%)	
African American	26 (32.91%)	23 (29.11%)	39 (50.00%)	
Other	5 (6.33%)	7 (8.86%)	3 (3.85%)	
Medical history				
Duration of HF, y	7.00 (3.00, 12.00)	6.00 (2.00, 11.00)	5.00 (2.00, 9.00)	0.15
Previous hospitalization for HF	65 (82.28%)	58 (73.42%)	65 (83.33%)	0.24
Time since last hospitalization for HF, y	0.42 (0.23, 2.09)	0.75 (0.26, 1.85)	0.43 (0.22, 1.62)	0.71
Missing	14	21	13	
Ejection fraction, %	27.05 (8.56)	26.28 (7.87)	26.60 (7.99)	0.84
Ischemic heart disease	42 (53.16%)	45 (56.96%)	37 (47.44%)	0.49
T2DM	57 (72.15%)	47 (59.49%)	46 (58.97%)	0.15
Atrial fibrillation	35 (44.30%)	31 (39.24%)	33 (42.31%)	0.81
ICD	47 (59.49%)	56 (70.89%)	41 (52.56%)	0.059
CRT				0.089
No	22 (46.81%)	37 (66.07%)	27 (65.85%)	
Yes	25 (53.19%)	19 (33.93%)	14 (34.15%)	
Missing	32	23	37	
Baseline HF/CV medications				
ACEI/ARB	50 (63.29%)	41 (51.90%)	39 (50.00%)	0.19
ARNI	21 (26.58%)	27 (34.18%)	25 (32.05%)	0.57
Beta-blocker	75 (94.94%)	77 (97.47%)	76 (97.44%)	0.74
Hydralazine	18 (22.78%)	10 (12.66%)	17 (21.79%)	0.20
Long-acting nitrates	28 (35.44%)	32 (40.51%)	20 (25.64%)	0.14
MRA	46 (58.23%)	55 (69.62%)	44 (56.41%)	0.18
Loop diuretics	67 (84.81%)	62 (78.48%)	71 (91.03%)	0.092
Digoxin	15 (18.99%)	20 (25.32%)	9 (11.54%)	0.085
Lipid-lowering agents∗	71 (89.87%)	64 (81.01%)	58 (74.36%)	0.041
Anticoagulant agent	33 (41.77%)	30 (37.97%)	29 (37.18%)	0.82
Glucose-lowering medications among patients with T2DM				
Insulin	35 (61.40%)	23 (48.94%)	19 (41.30%)	0.12
GLP-1RA	1 (1.75%)	1 (2.13%)	2 (4.35%)	0.69
DPP4-inhibitor	3 (5.26%)	9 (19.15%)	7 (15.22%)	0.087
Sulfonylurea∗	10 (17.54%)	17 (36.17%)	5 (10.87%)	0.008
Metformin	18 (31.58%)	18 (38.30%)	21 (45.65%)	0.34
Physical exam				
Body mass index, kg/m^2^	32.93 (6.78)	31.73 (6.09)	31.97 (7.04)	0.50
Missing	2	1	1	
Heart rate, beats/min	71.26 (12.34)	70.62 (11.16)	73.72 (13.35)	0.27
Missing	4	4	4	
Systolic blood pressure, mm Hg	122.83 (17.26)	123.81 (19.04)	126.31 (22.90)	0.53
Missing	0	0	1	
Baseline laboratory studies				
NT-proBNP, pg/mL	1,323.00 (715.00, 2,366.50)	1,056.00 (593.50, 2,183.00)	1,050.50 (592.25, 2,039.00)	0.42
BNP, pg/mL	304.00 (177.50, 616.50)	259.00 (131.75, 487.25)	242.00 (136.00, 485.00)	0.14
Missing	0	1	1	
eGFR, mL/min/1.73 m^2^	66.01 (19.26)	68.44 (21.46)	70.67 (23.62)	0.40
Urine albumin/creatinine ratio, mg/g	33.00 (7.00, 89.00)	17.00 (7.00, 79.00)	29.00 (5.75, 142.50)	0.69
Missing	6	8	10	
Hemoglobin A1c, %	7.07 (1.38)	7.15 (1.82)	7.01 (2.11)	0.89
Missing	1	0	0	
Hemoglobin, g/dL	13.14 (1.70)	13.50 (1.84)	13.70 (1.85)	0.15
Missing	0	2	2	
Functional measures				
NYHA class				0.82
II	53 (67.09%)	56 (70.89%)	52 (66.67%)	
III	26 (32.91%)	23 (29.11%)	26 (33.33%)	
KCCQ Overall Summary Score∗	62.95 (22.16)	72.35 (20.23)	65.05 (21.99)	0.017
KCCQ Clinical Summary Score∗	65.73 (23.05)	75.42 (19.32)	68.75 (22.08)	0.016
KCCQ Total Symptom Score∗	68.80 (24.66)	79.40 (19.91)	73.61 (22.99)	0.014
KCCQ Physical Limitation Score	62.65 (26.67)	71.08 (21.60)	63.89 (25.51)	0.072
Missing	0	1	0	
KCCQ Quality of Life Score	60.65 (23.76)	66.46 (24.31)	60.15 (25.79)	0.21
KCCQ Social Limitation Score∗	58.74 (27.97)	71.55 (25.84)	62.11 (29.14)	0.014
Missing	2	3	4	
6-min walk distance, m	279.23 (110.38)	300.46 (108.50)	311.74 (89.16)	0.14
Missing	1	0	0	
Weight (kg)	101.92 (24.69)	95.00 (22.92)	93.43 (22.23)	0.054

Values are absolute numbers (percentages and mean ± SD or median with IQR). ∗*P* < 0.05 for the difference between groups.

ACEI/ARB = angiotensin-converting enzyme inhibitor/angiotensin receptor blocker; ApoM = apolipoprotein M; ARNI = angiotensin receptor-neprilysin inhibitor; CRT = cardiac resynchronization therapy; CV = cardiovascular; DPP4 = dipeptidyl peptidase-4; eGFR = estimated glomerular filtration rate; GLP-1RA = glucagon-like peptide-1 receptor agonist; HF = heart failure; ICD = implantable cardioverter-defibrillator; KCCQ = Kansas City Cardiomyopathy Questionnaire; MRA = mineralocorticoid receptor antagonist; NT-proBNP = N-terminal pro B-type natriuretic peptide.

**Figure 1:**
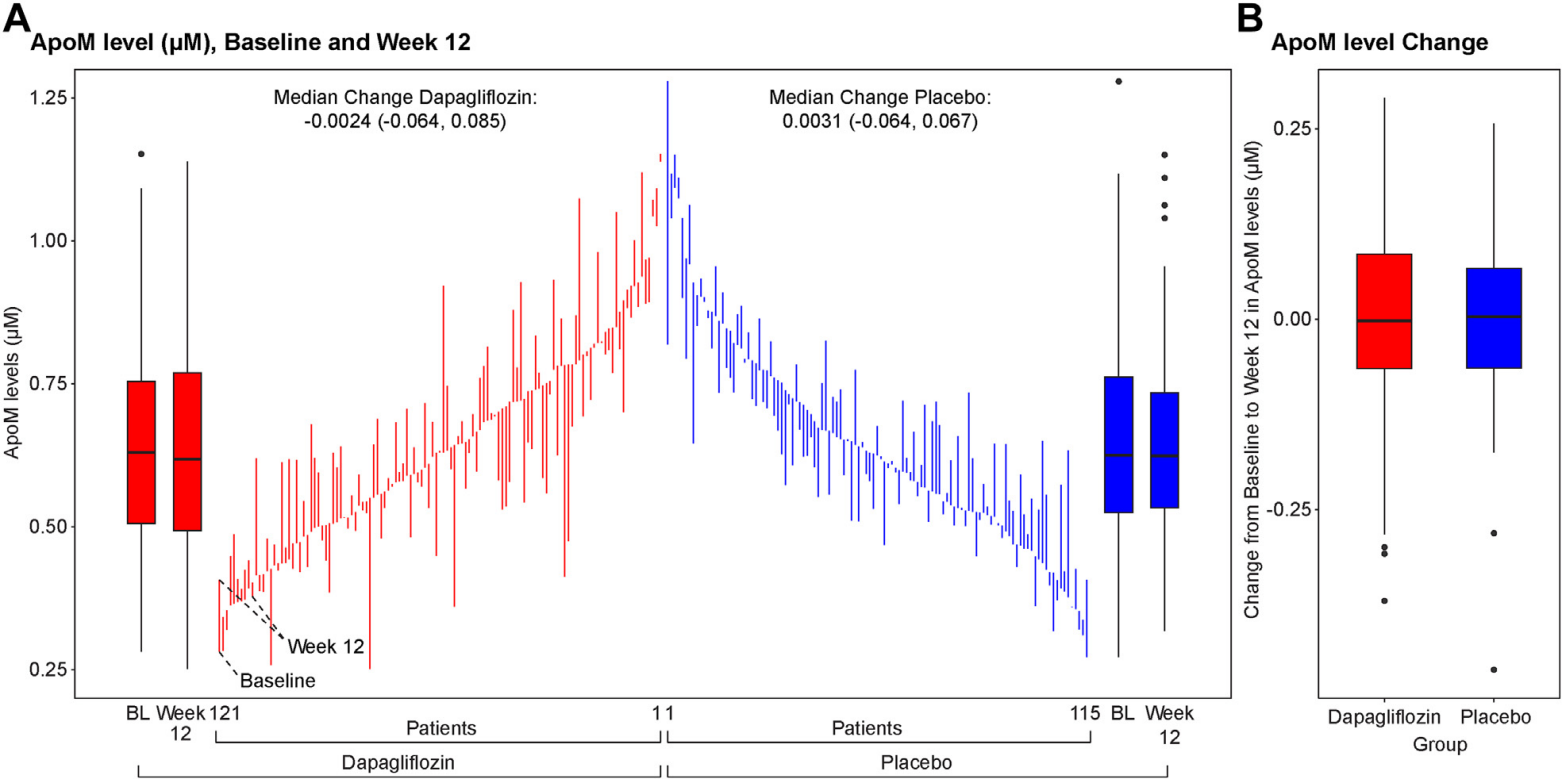
**Baseline and 12-Week Apolipoprotein M Levels in Dapagliflozin and Placebo Groups** (A) Individual patient-level ApoM values at baseline and 12 weeks are displayed for the dapagliflozin (red) and placebo (blue) groups. Vertical lines connecting baseline (BL) and 12-week values illustrate the within-patient change in ApoM levels over time. The median change (with IQR) for each group is displayed at the top of the figure. Box plots on the left and right summarize baseline and week 12 distributions. (B) Box plots represent the distribution of ApoM level changes from baseline to week 12 for each treatment group. Box edges indicate the IQR, with horizontal lines representing the median. Whiskers extend to the upper and lower adjacent values, and dots represent individual outliers. ApoM = apolipoprotein M.

**Figure 2:**
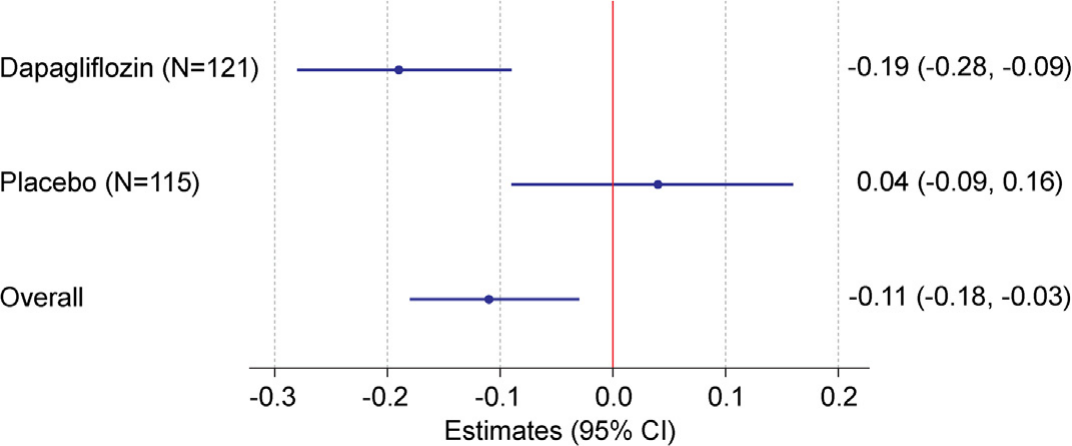
**Association Between Change in Apolipoprotein M and Log-Transformed N-Terminal Pro-B-Type Natriuretic Peptide at 12 Weeks in Dapagliflozin and Placebo Groups** This forest plot illustrates the estimated association between a 0.1 μM increase in ApoM and the corresponding change in log-transformed NT-proBNP at 12 weeks. Results are presented separately for the dapagliflozin group (N = 121), placebo group (N = 115), and overall cohort. Black dots represent the point estimates, with horizontal lines indicating the 95% CIs. The red vertical line at zero serves as a reference for no association. A statistically significant inverse association was observed in the dapagliflozin group (−0.19; 95% CI: −0.28 to −0.09), while the placebo group showed no significant association (0.04; 95% CI: −0.09 to 0.16). The overall cohort demonstrated a moderate inverse relationship (−0.11; 95% CI: −0.18 to −0.03). NT-proBNP = N-terminal pro-B-type natriuretic peptide; other abbreviation as in Figure 1.

Neither the dapagliflozin nor the placebo group had significant changes in ApoM levels from baseline to 12 weeks (t = 0.002; 95% CI: −0.019 to 0.024; *P* = 0.817 for the dapagliflozin group), (t = 0.003; 95% CI: −0.016 to 0.022; *P* = 0.753 for the placebo group), and there was no significant difference between dapagliflozin vs placebo on change in ApoM (−0.002 uM; 95% CI: −0.029 to 0.247; *P* = 0.89) (Figure 1). Both the mixed-effects model and GLM yielded consistent results, indicating that dapagliflozin had no statistically significant impact on ApoM levels (GLM: β = −0.00129; 95% CI: −0.0274 to 0.0248; *P* = 0.923; mixed-effects model: *P* = 0.703). However, after adjusting for baseline ApoM levels, age, race/ethnicity, sex, eGFR, and T2DM, each 0.1 uM increase in ApoM level at 12 weeks was associated with a significant decrease in log-transformed NT-proBNP in the overall cohort (β = −0.11; 95% CI: −0.18 to −0.03; *P* = 0.006) (Table 2). Moreover, this association varied between treatment groups (P for interaction = 0.025) and was more pronounced among patients randomized to dapagliflozin (β = −0.19; 95% CI: -0.28 to −0.09; *P* < 0.001) than the placebo group (β = 0.04; 95% CI: −0.09 to 0.16; *P* = 0.57) (Figure 2).

**Table 2:** The Relationship Between Change in ApoM Levels and Outcome Measures

Change in Outcomes	β-Coefficient (95% CI)^a^ per 0.1 μM Increase	*P* Value^b^	*P* Value for Interaction With Treatment Allocation^b^
Log NT-proBNP	−0.11 (−0.18 to −0.03)	0.006	0.025
6-min walk test in meters	3.54 (−4.39 to 11.47)	0.383	0.732
Weight in kilograms	−0.44 (−0.91 to 0.02)	0.064	0.720
KCCQ Overall Summary Score	1.38 (−0.42 to 3.19)	0.135	0.289
KCCQ Clinical Summary Score	1.04 (−0.70 to 2.77)	0.244	0.345
KCCQ Total Symptom Score	1.32 (−0.64 to 3.28)	0.189	0.344
KCCQ Physical Limitation Score	0.69 (−1.53 to 2.92)	0.541	0.494
KCCQ Quality of Life Score	0.95 (−1.47 to 3.37)	0.444	0.330
KCCQ Social Limitation Score	2.18 (−0.62 to 4.99)	0.129	0.400

Abbreviations as in Table 1.

a Adjusting for baseline value of the respective outcome, baseline ApoM/S1P levels, age, race/ethnicity, sex, eGFR, and T2DM (T2DM).

b Adjusting for treatment allocation and the interactions by treatment allocation.

The characteristics of patients were also presented by groups defined by the relationship between changes in ApoM and change in log-transformed NT-proBNP (Supplemental Table 2). An increase in ApoM was defined as a change greater than the median change observed (>0.00159 μM). Likewise, an increase in log-transformed NT-proBNP was defined as a change greater than the median change observed (>0.692 μM). Patients were categorized into 4 groups based on their ApoM and log-transformed NT-proBNP changes: decrease-decrease, decrease-increase, increase-decrease, and increase-increase. The patients for whom ApoM increased over 12 weeks while log-transformed NT-proBNP decreased (increase-decrease) presented with greater UACR at baseline than all other subgroups (33 mg/g [10.00, 100.00] compared to 14 mg/g [5.00, 57.00], 25 mg/g [7.00, 171.00], and 16 mg/g [6.00, 76.00] for the other subgroups) (Table 3). This group also experienced the most significant decrease in UACR after 12 weeks when contrasted with the other subgroups (−5 mg/g compared to 0.0, 1.0, and 0.0 in other subgroups) (Table 3).

**Table 3:** Change in UACR After 12 Weeks by the Change in ApoM and Log-Transformed NT-proBNP Subgroups

Urine Albumin/Creatinine Ratio, mg/g	Decrease-Decrease (n = 56)	Decrease-Increase (n = 62)	Increase-Decrease (n = 62)	Increase-Increase (n = 56)
At baseline	14.00 (5.00, 57.00)	25.00 (7.00, 171.00)	33.00 (10.00, 100.00)	16.00 (6.00, 76.00)
Missing	1	1	1	2
At 12 wk	13.00 (5.25, 42.00)	23.00 (9.75, 108.50)	17.00 (7.00, 41.00)	16.00 (5.75, 77.25)
Missing	2	2	1	0
Change	0.00 (−14.00, 8.00)	1.00 (−15.00, 18.00)	−5.00 (−68.00, 4.00)	1.0 (−11.50, 11.75)
Missing	3	3	2	2

Urine albumin-to-creatinine ratio (UACR) values are presented as median (IQR, Q1–Q3) in mg/g at baseline and 12 weeks, stratified by change in ApoM and log-transformed NT-proBNP subgroups. The numbers in parentheses indicate the IQR of the variable distribution. An increase in ApoM was defined as a change greater than the median change observed (>0.00159), and for log NT-pro BNP, an increase was defined as a change greater than the median change observed (>0.692). A decrease in ApoM was defined as a change less than the median change observed (<0.00159), and for log NT-proBNP, a decrease was defined as a change less than the median change observed (<0.692). Patients were categorized into 4 groups based on this: Decrease-Decrease, Decrease-Increase, Increase-Decrease, and Increase-Increase. Specifically, Increase-Increase is defined as an increase in ApoM coinciding with an increase in log NT-proBNP, compared to median change; Decrease-Increase is defined as a decrease in ApoM coinciding with an increase in log NT-proBNP, compared to the median change; Increase-Decrease is defined as an increase in ApoM coinciding with a decrease in log NT-proBNP, compared to the median change; and Increase-Increase is defined as an increase in ApoM coinciding with an increase in NT-proBNP, compared to median change.

UACR = urine albumin-creatinine ratio; other abbreviations as in Table 1.

**Figure 3:**
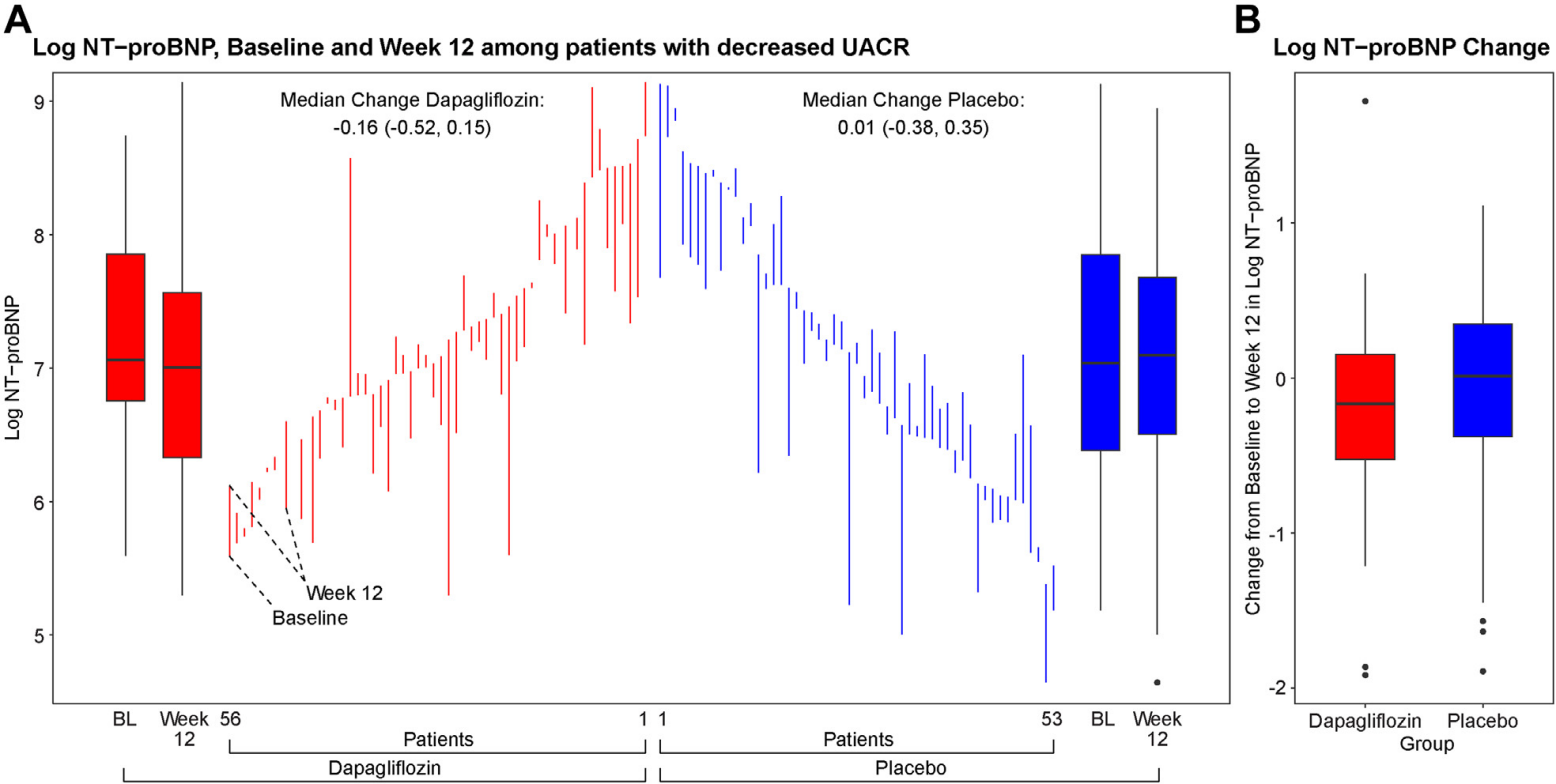
**Change in Log-Transformed N-Terminal Pro-B-Type Natriuretic Peptide From Baseline to Week 12 Among Patients With Decreased Urine Albumin-Creatinine Ratio** (A) Individual patient-level log NT-proBNP values at baseline (BL) and 12 weeks are displayed for the dapagliflozin (red) and placebo (blue) groups, specifically among patients who experienced a decrease in UACR over 12 weeks. Vertical lines connecting baseline and week 12 values illustrate within-patient changes in NT-proBNP over time. The median change (with IQR) for each group is displayed at the top of the figure. Box plots on the left and right summarize the baseline and week 12 distributions. (B) Box plots represent the distribution of NT-proBNP level changes from baseline to week 12 for each treatment group. Box edges indicate the IQR, with horizontal lines representing the median. Whiskers extend to the upper and lower adjacent values, and dots represent individual outliers. UACR = urine albumin-creatinine ratio; other abbreviation as in Figure 2.

Figure 3 demonstrates the relationship between the changes in log-transformed NT-proBNP for patients treated with dapagliflozin or placebo who experienced a decrease in UACR after 12 weeks. The inverse relationship between the change in ApoM and the change in log NT-proBNP varied by UACR change (*P* for interaction = 0.028) and was more pronounced among patients whose UACR declined after 12 weeks (95% CI: −0.312 to −0.104; *P* < 0.001). Among patients whose UACR declined, the association between the change in ApoM and the change in log-transformed NT-proBNP was more pronounced among those treated with dapagliflozin (95% CI: −0.407 to −0.146; *P* < 0.001; *P* for interaction <0.001). Dapagliflozin-treated patients with a reduction in UACR at 12 weeks (n = 53, 22%) experienced a mean decrease in log-transformed NT-proBNP of −0.28 per 0.1 μM increase in ApoM (95% CI: −0.41 to −0.15; *P* < 0.001); vs −0.07 (95% CI: −0.19 to −0.06; *P* = 0.47) for dapagliflozin-treated patients without a change or increase in UACR. In contrast, placebo-treated patients with reduced UACR over 12 weeks did not have a significant reduction in log-transformed NT-proBNP per 0.1 μM increase in ApoM (β = −0.17; 95% CI: −0.37 to 0.035; *P* = 0.11).

## Discussion

In the DEFINE-HF double-blind, randomized, placebo-controlled clinical trial of patients with HFrEF, patients treated with dapagliflozin did not experience statistically significant changes in ApoM compared to placebo. However, we observed a significant interaction between dapagliflozin randomization and changes in ApoM and NT-proBNP, indicating favorable changes in cardiac biomarkers coinciding with dapagliflozin therapy. Furthermore, patients who experienced the greatest decrease in UACR over the course of the study were precisely the ones who had increases in ApoM and decreases in log-transformed NT-proBNP. Moreover, this observation was most pronounced in those treated with dapagliflozin.

Several potential explanations exist for why treatment with dapagliflozin did not result in significant differences in ApoM compared to placebo. Firstly, ApoM values were measured at baseline and 12 weeks, whereas prior animal studies examining this relationship repeated measurements in days rather than weeks. Also, animal studies have shown that dapagliflozin preserves ApoM levels in acute inflammation through reabsorption in the proximal tubule of the kidney rather than through up-regulation of ApoM synthesis in the liver or kidney.^11^ Thus, patients without active inflammation before treatment with dapagliflozin may not experience significant changes in ApoM after treatment with dapagliflozin compared to placebo. The median UACR being normal at 18 mg/g in DEFINE-HF patients may provide insight into why the general population of dapagliflozin-treated patients in this study did not experience substantial differences in ApoM levels. In contrast, those with the highest UACR treated with dapagliflozin were the most likely to experience an increase in ApoM levels associated with a decrease in NT-proBNP.

Patients with intrinsic kidney injury, indicated by an elevated UACR, may derive significant benefits from dapagliflozin therapy for 2 key reasons. First, they are at heightened risk of adverse clinical events.^16^ Second, UACR may serve as a crucial determinant of response to dapagliflozin treatment. The DECLARE-TIMI 58 (Dapagliflozin Effect on Cardiovascular Events-Thrombolysis In Myocardial Infarction 58) trial supported this hypothesis, demonstrating that dapagliflozin significantly reduced UACR levels compared to placebo, irrespective of baseline UACR and eGFR, reinforcing its renoprotective effects.^17^ Notably, all baseline UACR subgroups—except those with levels <15 mg/g—experienced a substantial decline in UACR within 6 months of treatment, aligning with our findings. Our study further suggests that patients with UACR >25 mg/g may represent a distinct responder group to dapagliflozin (n = 103, 44%). In these individuals, dapagliflozin treatment was associated with an increase in ApoM levels, which correlated with a reduction in NT-proBNP, reinforcing a potential ApoM-mediated mechanism linking dapagliflozin to improved cardiac and renal outcomes (Central Ilustration). Prior preclinical studies suggest that dapagliflozin preserves ApoM levels by mitigating endothelial permeability changes and inflammation through the S1P signaling pathway, preventing its urinary loss in the setting of kidney dysfunction. These findings highlight a possible mechanistic link between dapagliflozin, ApoM, and natriuretic peptide regulation, warranting further investigation into ApoM as a biomarker of SGLT2i response.

**Central Illustration:**
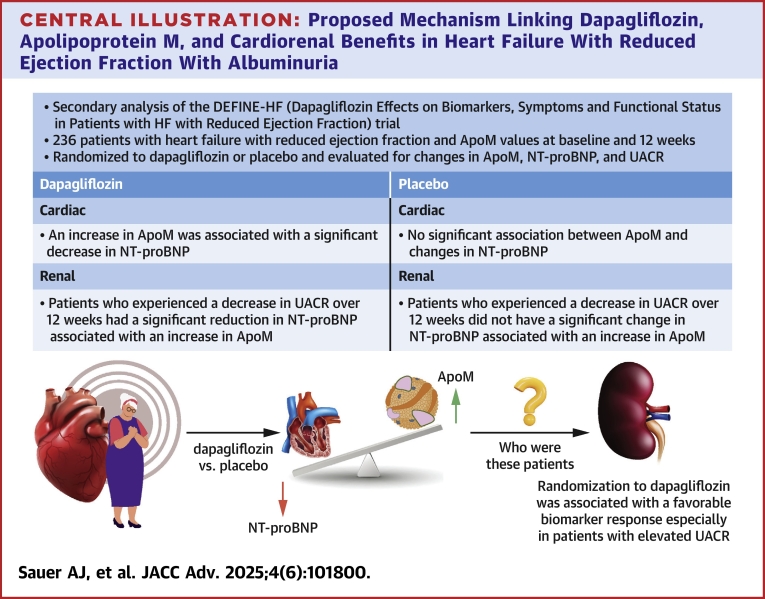
**Proposed Mechanism Linking Dapagliflozin, Apolipoprotein M, and Cardiorenal Benefits in Heart Failure With Reduced Ejection Fraction With Albuminuria** In patients with albuminuria (urinary albumin-to-creatinine ratio [UACR] >25 mg/g), treatment with dapagliflozin was associated with an inverse relationship between ApoM and N-terminal pro-B-type natriuretic peptide (NT-proBNP), suggesting a potential dapagliflozin-mediated effect. This relationship may be mediated by ApoM-related protective mechanisms, including enhanced endothelial cell chemotaxis, wound healing, and angiogenesis via the sphingosine-1-phosphate (S1P) signaling pathway, which contribute to favorable cardiac remodeling and the attenuation of kidney fibrosis and nephropathy. The significant interaction with dapagliflozin supports further investigation into ApoM as a potential mediator of its cardiorenal benefits. HFrEF = heart failure with reduced ejection fraction; other abbreviations as in Figures 1 to 3.

Similarly, EMPA-REG OUTCOME (Empagliflozin Cardiovascular Outcome Event Trial in Type 2 Diabetes Mellitus Patients) showed that patients with higher UACR levels at baseline experienced more significant reductions in UACR after treatment with another SGLT2i, empagliflozin.^18^ Several studies support the idea that groups with higher UACR levels benefit more from SGLT2i than groups with milder UACR elevation;^17^^,^^19^^,^^20^ however, how these benefits occur and the relationship between kidney injury and ApoM is yet to be determined. Future studies may allow for further investigation into the role of ApoM and its exact effects on kidney injury and vice versa. Additionally, a higher baseline UACR was found to have a significant positive association with NT-proBNP in patients with HF in the BIOSTAT-CHF (Biology Study to Tailored Treatment in Chronic Heart Failure) trial, suggesting that increases in the severity of albuminuria are associated with increases in adverse biomarker changes. The BIOSTAT-CHF trial proposed endothelial dysfunction and loss of glycocalyx integrity as the underlying mechanisms for the association between albuminuria and HF.^21^ Interestingly, the loss of glycocalyx integrity within the renal endothelium supports our findings that patients with high UACR levels at baseline exhibit favorable changes in ApoM and NT-pro-BNP after dapagliflozin randomization, possibly due to SGLT2i's protective effects on the renal endothelium.^11^ Additionally, the role of SGLT2i in favorable cardiac remodeling in patients with high UACR is supported by the CREDENCE (Canagliflozin and Renal Endpoints in Diabetes with Established Nephropathy Clinical Evaluation) trial, which found that treatment with the SGLT2i canagliflozin decreased NT-proBNP by 15% after 1 year in patients with T2DM and CKD with significant albuminuria, suggesting favorable cardiac remodeling mediated by dapagliflozin in the setting of kidney injury.^22^ Our prior murine studies have suggested that dapagliflozin's anti-inflammatory and cardioprotective effects rely on an ApoM-mediated pathway, which reduces endothelial permeability by preserving circulating ApoM levels.^11^ Animal studies have shown ApoM to accomplish these anti-inflammatory and cardioprotective effects through its involvement in protecting against lysosomal injury and autophagy impairment.^8^

The observed interaction between ApoM, NT-pro-BNP, and dapagliflozin therapy is consistent with unique biology related to SGLT2i since, if ApoM were merely a marker of inflammation in HF patients, we would expect to observe a similar inverse relationship with NT-pro-BNP in patients randomized to placebo. In animal studies, ApoM protects against kidney disease in mice by attenuating kidney fibrosis and injury and suppressing nephropathy.^9^^,^^23^ SGLT2i may participate in this effect by preventing the urinary loss of ApoM, as shown in lipopolysaccharide (LPS) murine models, and this potential mechanism should be further researched in human patients as well. While LPS alone leads to reductions in ApoM, dapagliflozin treatment interferes with this effect. It attenuates the ApoM decline by enhancing ApoM reabsorption in the kidney, a process mediated by the protein receptor megalin.^11^ Furthermore, dapagliflozin was found to attenuate LPS-induced endothelial leak and inflammation in an ApoM-dependent manner.

DEFINE-HF is the first clinical trial to investigate the relationship between SGLT2i, ApoM, and UACR in human subjects. Our results validate those of previous studies regarding the kidney-protective effects of SGLT2i. DAPA-CKD (Dapagliflozin in Patients with CKD) assessed the impact of dapagliflozin on a primary composite outcome which consisted of a 50% reduction in eGFR, onset of end-stage kidney disease and death from renal or cardiovascular causes in patients with CKD with or without type II diabetes. DAPA-CKD reported that patients randomized to dapagliflozin had a lower risk for the primary outcome.^24^ Compared to DAPA-CKD, DEFINE-HF enrolled a U.S. population with a higher proportion of Black patients (37.6% vs 4.7%), a dramatically lower UACR median (18 mg/g vs 949 mg/g), and a much shorter follow-up period (12 weeks vs 2.4 years). EMPEROR (Empagliflozin Outcome Trial in Patients with Chronic Heart Failure and a Reduced or Preserved Ejection Fraction)-Reduced^25^ and EMPEROR-Preserved^26^ trials assessed the effects of empagliflozin on major renal events in patients with HF with a baseline ejection fraction of 40% or less in EMPEROR-Reduced and 40% or more in EMPEROR-Preserved. These trials reported that treatment with empagliflozin was associated with a lower incidence of new microalbuminuria and an increase in the remission rate to sustained normoalbuminuria or microalbuminuria. Compared to EMPEROR-Reduced and Preserved, DEFINE-HF enrolled patients with similar UACR levels at baseline (18 mg/g vs 15 and 36 mg/g in patients with and without CKD EMPEROR-Reduced and 30 and 15 mg/g in patients with and without diabetes in EMPEROR-Preserved).

### Study limitations

The present study has multiple strengths, including the study of the effect of dapagliflozin on ApoM levels and the associations between ApoM, NT-proBNP, and UACR in an appropriately powered randomized clinical trial involving patients with HFrEF. Several limitations should be considered when interpreting our findings. The 12-week study duration limited insights into the long-term effects of dapagliflozin on ApoM and UACR levels. Additionally, the sample size, while sufficient for overall analyses, may have reduced statistical power in stratified analyses, particularly those evaluating the interaction between ApoM, NT-proBNP, and UACR subgroups. These exploratory subgroup analyses should be interpreted with caution, and larger studies will be needed to confirm these findings. Furthermore, the study population included only U.S.-based patients, which may limit the generalizability of our findings. Additionally, most participants had only a mild degree of CKD, as indicated by relatively normal UACR levels at baseline, which may have limited our ability to observe significant changes in ApoM after dapagliflozin treatment. Future studies should include patients with higher baseline UACR levels and a more extended follow-up period to assess better the impact of dapagliflozin on ApoM and its potential role in cardiorenal protection. By addressing these limitations, future research can build upon our findings to further elucidate the mechanistic pathways linking SGLT2 inhibition, ApoM, and kidney and cardiovascular outcomes.

## Conclusions

This Secondary analysis of DEFINE-HF demonstrates that while dapagliflozin treatment did not result in significant overall changes in ApoM levels, an inverse association between ApoM and NT-proBNP was observed, particularly in dapagliflozin-treated patients. This relationship was most pronounced in individuals with albuminuria, suggesting that dapagliflozin may play a role in preserving ApoM levels in the setting of kidney dysfunction. While some NT-proBNP reductions were also observed in the placebo group, the significant interaction between treatment allocation and the ApoM-NT-proBNP association supports a potential dapagliflozin-mediated effect. These findings highlight the need for further research to clarify the mechanistic pathways underlying the interplay between ApoM, kidney function, and natriuretic peptide regulation in HF.

## Funding support and author disclosures

DEFINE-HF was an investigator-initiated trial funded by 10.13039/100004325AstraZeneca Pharmaceuticals LP (Wilmington, DE) and conducted by Saint Luke's Mid America Heart Institute (Kansas City, MO) independent of the funding source. AstraZeneca AB (Mölndal, Sweden) provided funding to Washington University in St. Louis (St. Louis, MO) for the ApoM testing. Dr Sauer has received grant/research support from 10.13039/100004325AstraZeneca, CSL Vifor, Rivus, 10.13039/100004319Pfizer, 10.13039/100004326Bayer, and 10.13039/100001003Boehringer Ingelheim; has received honoraria from Bayer, Abbott, Impulse Dynamics, Boston Scientific, Medtronic, Edwards Lifesciences, Biotronik, General Prognostics, Story Health, and Acorai; and has stock ownership with ISHI. Dr Kosiborod has received grant/research support from 10.13039/100004325AstraZeneca, 10.13039/100001003Boehringer Ingelheim, and 10.13039/100004319Pfizer; has received honoraria from AstraZeneca, Boehringer Ingelheim, and Novo Nordisk; has served as a consultant for 35Pharma, Alnylam, Amgen, Applied Therapeutics, Arrowhead Pharmaceuticals, AstraZeneca, Bayer, Boehringer Ingelheim, Corcept Therapeutics, Cytokinetics, Dexcom, Eli Lilly, Esperion Therapeutics, Imbria Pharmaceuticals, Janssen, Lexicon, Merck (Diabetes and Cardiovascular), Novo Nordisk, Pfizer, Pharmacosmos, Regeneron, Roche, Sanofi, scPharmaceuticals, Structure Therapeutics, Vifor Pharma, and Youngene Therapeutics; has received other research support (data analytic center fees) from AstraZeneca and Vifor Pharma; has stock options with Artera Health and Saghmos Therapeutics; and reports employment by AstraZeneca R&D, effective January 6, 2025. Dr Javaheri has received grant/research support from 10.13039/100004325AstraZeneca and Bitterroot Bio; has advisory board and ownership interest for Mobius Scientific; and holds patents on ApoM fusion-protein nanodiscs for treatment of HF and eye diseases. Dr Inzucchi has received grant/research support from NIDDK; has served as a consultant/advisor for AstraZeneca, Boehringer Ingelheim, Novo Nordisk, Bayer, Merck, and Pfizer. All other authors have reported that they have no relationships relevant to the contents of this paper to disclose.
