# Supplementary material for: Dapagliflozin's Association With Cardiorenal Outcomes and Apolipoprotein M Levels in HFrEF Patients: Insights From DEFINE-HF
Source: JACC Adv. 2025 Jun 25;4(6):101800. doi: 10.1016/j.jacadv.2025.101800 (PMC12277613; doi:10.1016/j.jacadv.2025.101800)
Supplement: Supplemental Data [file mmc1.docx]

**Supplemental Table 1. Characteristics of patients included and excluded from the study^a^**

| **Characteristic** | **The biomarker subset**  **N = 236** | **The exclusion subset,**  **N = 27** | **p-value^b^** |
| --- | --- | --- | --- |
| **Treatment group** | | | |
| Placebo | 115 (48.73%) | 17 (62.96%) | 0.16 |
| Dapagliflozin | 121 (51.27%) | 10 (37.04%) |  |
| **Demographics** | | | |
| Age, y | 61.97 (11.05) | 55.48 (14.04) | 0.027 |
| Male | 177 (75.00%) | 16 (59.26%) | 0.080 |
| Race |  |  | 0.054 |
| White | 133 (56.36%) | 11 (40.74%) |  |
| African American | 88 (37.29%) | 11 (40.74%) |  |
| Other | 15 (6.36%) | 5 (18.52%) |  |
| **Medical history** | | | |
| Duration of HF, y (median Q1, Q3) | 6.00 (2.00, 11.00) | 4.00 (2.00, 11.50) | 0.96 |
| Previous hospitalization for HF | 188 (79.66%) | 21 (77.78%) | 0.82 |
| Time since last hospitalization for HF, y (median Q1, Q3) | 0.52 (0.23, 1.83) | 0.38 (0.21, 1.07) | 0.36 |
| Missing | 48 | 6 |  |
| Ejection fraction, % | 26.64 (8.12) | 24.72 (8.09) | 0.25 |
| Ischemic heart disease | 124 (52.54%) | 15 (55.56%) | 0.77 |
| T2DM | 150 (63.56%) | 16 (59.26%) | 0.66 |
| Atrial Fibrillation | 99 (41.95%) | 7 (25.93%) | 0.11 |
| ICD | 144 (61.02%) | 19 (70.37%) | 0.34 |
| CRT |  |  | 0.30 |
| No | 86 (59.72%) | 9 (47.37%) |  |
| Yes | 58 (40.28%) | 10 (52.63%) |  |
| Missing | 92 | 8 |  |
| **Baseline HF/CV medications** | | | |
| ACEI/ARB | 130 (55.08%) | 11 (40.74%) | 0.16 |
| ARNI | 73 (30.93%) | 12 (44.44%) | 0.16 |
| ß-blocker | 228 (96.61%) | 26 (96.30%) | >0.99 |
| Hydralazine | 45 (19.07%) | 0 (0.00%) | 0.006 |
| Long-acting nitrates | 80 (33.90%) | 6 (22.22%) | 0.22 |
| MRA | 145 (61.44%) | 15 (55.56%) | 0.55 |
| Loop diuretics | 200 (84.75%) | 25 (92.59%) | 0.39 |
| Digoxin | 44 (18.64%) | 2 (7.41%) | 0.19 |
| Lipid-lowering agents | 193 (81.78%) | 18 (66.67%) | 0.062 |
| Anticoagulant agent | 92 (38.98%) | 8 (29.63%) | 0.34 |
| **Glucose lowering medications among patients with T2DM** | | | |
| Insulin | 77 (51.33%) | 10 (62.50%) | 0.40 |
| GLP-1RA | 4 (2.67%) | 1 (6.25%) | 0.40 |
| DPP4-inhibitor | 19 (12.67%) | 2 (12.50%) | >0.99 |
| Sulfonylurea | 32 (21.33%) | 3 (18.75%) | >0.99 |
| Metformin | 57 (38.00%) | 5 (31.25%) | 0.60 |
| **Physical exam** | | | |
| Body mass index | 32.21 (6.64) | 31.77 (7.06) | 0.76 |
| Missing | 4 | 1 |  |
| Heart rate^c^ | 71.86 (12.33) | 71.83 (12.61) | >0.99 |
| Missing | 12 | 2 |  |
| Systolic blood pressure^c^ | 124.30 (19.82) | 118.65 (23.26) | 0.23 |
| Missing | 1 | 0 |  |
| **Baseline laboratory studies** | | | |
| NT-proBNP, pg/mL (median Q1, Q3) | 1,118.00 (615.00, 2,193.25) | 1,610.00 (699.50, 2,421.50) | 0.58 |
| BNP, pg/mL (median Q1, Q3) | 265.00 (141.50, 553.50) | 396.50 (147.75, 681.75) | 0.43 |
| Missing | 2 | 1 |  |
| eGFR, mL/min/1.73m^2^ | 68.36 (21.50) | 75.19 (27.13) | 0.22 |
| Urine albumin/creatinine ratio, mg/g (median Q1, Q3) | 18.00 (6.00, 88.00) | 19.00 (6.50, 55.00) | 0.59 |
| Missing | 5 | 0 |  |
| Hemoglobin A1c, % | 7.08 (1.79) | 7.97 (2.81) | 0.12 |
| Missing | 1 | 0 |  |
| Hemoglobin, g/dL | 13.45 (1.80) | 13.13 (1.99) | 0.45 |
| Missing | 5 | 0 |  |
| **Functional measures** | | | |
| NYHA Class |  |  | 0.014 |
| Class II | 161 (68.22%) | 12 (44.44%) |  |
| Class III | 75 (31.78%) | 15 (55.56%) |  |
| KCCQ Overall Summary Score | 66.79 (21.76) | 70.80 (18.81) | 0.31 |
| KCCQ Clinical Summary Score | 69.97 (21.83) | 73.65 (19.73) | 0.37 |
| KCCQ Total Symptom Score | 73.94 (22.92) | 74.96 (21.03) | 0.81 |
| KCCQ Physical Limitation Score | 65.86 (24.87) | 72.35 (22.10) | 0.16 |
| Missing | 1 | 0 |  |
| KCCQ Quality of Life Score | 62.43 (24.69) | 65.43 (22.49) | 0.52 |
| KCCQ Social Limitation Score | 64.12 (28.08) | 70.45 (25.07) | 0.23 |
| Missing | 9 | 0 |  |
| 6-minute walk distance, meters | 297.16 (103.59) | 299.55 (91.86) | 0.90 |
| Missing | 1 | 1 |  |
| Weight (kilograms) | 96.80 (23.50) | 95.23 (20.16) | 0.71 |
| BMI indicates body mass index; eGFR, estimated glomerular filtration rate; KCCQ, Kansas City Cardiomyopathy Questionnaire; NYHA, New York Heart Association; NT-proBNP, N-terminal pro B-type natriuretic peptide. Values are shown as absolute numbers (percentages and mean ± SD or median with IQR).  ^a^ Mean (SD) or Median (IQR); n (%)  ^b^ Welch Two Sample t-test or Wilcoxon rank sum test; Pearson's Chi-squared test or Fisher's exact test  ^c^ Blood pressure and heart rate measured from noninvasive cuff measurements for patients in sinus rhythm and manual pulse and blood pressure for patients in atrial fibrillation | | | |

**Supplemental Table 2. Patient characteristics stratified by 12-week change in ApoM and log-transformed NT-proBNP subgroups**

|  | **Change in ApoM and change in log NT-proBNP^a^** | | | |
| --- | --- | --- | --- | --- |
| **Characteristic** | **Decrease-Decrease**  **N = 56** | **Decrease-Increase**  **N = 62** | **Increase-Decrease**  **N = 62** | **Increase-Increase**  **N = 56** |
| **Treatment group** | | | | |
| Dapagliflozin | 27 (48.21%) | 35 (56.45%) | 36 (58.06%) | 23 (41.07%) |
| Placebo | 29 (51.79%) | 27 (43.55%) | 26 (41.94%) | 33 (58.93%) |
| **ApoM/S1P levels (uM) at baseline** | 0.69 (0.19) | 0.70 (0.17) | 0.58 (0.18) | 0.60 (0.16) |
| **Demographics** |  |  |  |  |
| Age, y | 61.48 (11.34) | 60.90 (11.33) | 61.34 (11.76) | 64.34 (9.46) |
| Male | 45 (80.36%) | 47 (75.81%) | 47 (75.81%) | 38 (67.86%) |
| Race |  |  |  |  |
| White | 36 (64.29%) | 35 (56.45%) | 32 (51.61%) | 30 (53.57%) |
| African American | 19 (33.93%) | 26 (41.94%) | 24 (38.71%) | 19 (33.93%) |
| Other | 1 (1.79%) | 1 (1.61%) | 6 (9.68%) | 7 (12.50%) |
| **Medical history** | | | | |
| Duration of HF, y (median Q1, Q3) | 5.50 (2.75, 9.25) | 7.00 (3.00, 11.00) | 6.00 (2.00, 11.00) | 4.00 (1.00, 10.25) |
| Previous hospitalization for HF |  |  |  |  |
| No | 9 (16.07%) | 19 (30.65%) | 8 (12.90%) | 12 (21.43%) |
| Yes | 47 (83.93%) | 43 (69.35%) | 54 (87.10%) | 44 (78.57%) |
| Time since last hospitalization for HF, y (median Q1, Q3) | 0.77 (0.33, 2.59) | 0.49 (0.25, 1.26) | 0.40 (0.22, 1.57) | 0.55 (0.22, 1.93) |
| Missing | 9 | 19 | 8 | 12 |
| Ejection fraction, % | 25.69 (8.16) | 26.89 (8.12) | 26.16 (8.16) | 27.87 (8.07) |
| Ischemic heart disease | 29 (51.79%) | 35 (56.45%) | 28 (45.16%) | 32 (57.14%) |
| T2DM | 35 (62.50%) | 41 (66.13%) | 39 (62.90%) | 35 (62.50%) |
| Atrial Fibrillation | 31 (55.36%) | 27 (43.55%) | 22 (35.48%) | 19 (33.93%) |
| ICD | 37 (66.07%) | 38 (61.29%) | 31 (50.00%) | 38 (67.86%) |
| CRT |  |  |  |  |
| No | 22 (59.46%) | 26 (68.42%) | 17 (54.84%) | 21 (55.26%) |
| Yes | 15 (40.54%) | 12 (31.58%) | 14 (45.16%) | 17 (44.74%) |
| Missing | 19 | 24 | 31 | 18 |
| **Baseline HF/CV medications** | | | | |
| ACEI/ARB | 34 (60.71%) | 28 (45.16%) | 37 (59.68%) | 31 (55.36%) |
| ARNI | 15 (26.79%) | 22 (35.48%) | 18 (29.03%) | 18 (32.14%) |
| ß-blocker | 53 (94.64%) | 60 (96.77%) | 61 (98.39%) | 54 (96.43%) |
| Hydralazine | 8 (14.29%) | 12 (19.35%) | 15 (24.19%) | 10 (17.86%) |
| Long-acting nitrates | 15 (26.79%) | 20 (32.26%) | 23 (37.10%) | 22 (39.29%) |
| MRA | 36 (64.29%) | 35 (56.45%) | 41 (66.13%) | 33 (58.93%) |
| Loop diuretics | 47 (83.93%) | 54 (87.10%) | 54 (87.10%) | 45 (80.36%) |
| Digoxin | 13 (23.21%) | 9 (14.52%) | 9 (14.52%) | 13 (23.21%) |
| Lipid-lowering agents | 43 (76.79%) | 49 (79.03%) | 51 (82.26%) | 50 (89.29%) |
| Anticoagulant agent | 30 (53.57%) | 24 (38.71%) | 19 (30.65%) | 19 (33.93%) |
| **Glucose lowering medications among patients with T2DM** | | | | |
| Insulin | 16 (45.71%) | 21 (51.22%) | 23 (58.97%) | 17 (48.57%) |
| GLP-1RA | 2 (5.71%) | 1 (2.44%) | 0 (0.00%) | 1 (2.86%) |
| DPP4-inhibitor | 4 (11.43%) | 6 (14.63%) | 4 (10.26%) | 5 (14.29%) |
| Sulfonylurea | 5 (14.29%) | 12 (29.27%) | 10 (25.64%) | 5 (14.29%) |
| Metformin | 12 (34.29%) | 15 (36.59%) | 20 (51.28%) | 10 (28.57%) |
| **Physical exam** | | | | |
| Body mass index | 33.03 (6.54) | 32.10 (6.58) | 32.35 (7.46) | 31.36 (5.83) |
| Missing | 1 | 2 | 0 | 1 |
| Heart rate^b^ | 71.54 (12.38) | 74.83 (13.04) | 71.68 (12.87) | 68.94 (10.22) |
| Missing | 5 | 1 | 3 | 3 |
| Systolic blood pressure^b^ | 124.06 (17.26) | 123.35 (19.94) | 121.50 (18.74) | 128.70 (22.77) |
| Missing | 1 | 0 | 0 | 0 |
| **Baseline laboratory studies** | | | | |
| NT-proBNP, pg/mL (median Q1, Q3) | 1,168.50 (711.00, 1,999.25) | 922.50 (527.00, 1,958.50) | 1,651.50 (924.25, 3,326.75) | 887.50 (511.25, 1,718.00) |
| BNP, pg/mL (median Q1, Q3) | 250.00 (139.00, 461.00) | 244.50 (134.25, 418.50) | 376.00 (205.50, 752.75) | 210.00 (101.50, 485.00) |
| Missing | 1 | 0 | 0 | 1 |
| eGFR, mL/min/1.73m^2^ | 66.80 (23.54) | 69.97 (19.67) | 69.20 (24.45) | 67.21 (17.94) |
| Urine albumin/creatinine ratio, mg/g (median Q1, Q3) | 14.00 (5.00, 57.00) | 25.00 (7.00, 171.00) | 33.00 (10.00, 100.00) | 16.00 (6.00, 76.00) |
| Missing | 8 | 6 | 6 | 4 |
| Hemoglobin A1c, % | 7.06 (1.79) | 7.28 (2.12) | 7.14 (1.67) | 6.81 (1.52) |
| Missing | 1 | 0 | 0 | 0 |
| Hemoglobin, g/dL | 13.70 (1.78) | 13.73 (2.01) | 13.32 (1.71) | 13.03 (1.63) |
| Missing | 1 | 2 | 0 | 1 |
| **Functional measures** | | | | |
| NYHA Class |  |  |  |  |
| Class II | 41 (73.21%) | 39 (62.90%) | 43 (69.35%) | 38 (67.86%) |
| Class III | 15 (26.79%) | 23 (37.10%) | 19 (30.65%) | 18 (32.14%) |
| KCCQ Overall Summary Score | 68.11 (22.08) | 67.09 (20.01) | 63.78 (22.77) | 68.46 (22.41) |
| KCCQ Clinical Summary Score | 70.91 (23.00) | 70.16 (20.74) | 67.00 (23.01) | 72.11 (20.67) |
| KCCQ Total Symptom Score | 74.91 (23.58) | 75.29 (21.99) | 70.03 (24.94) | 75.82 (20.96) |
| KCCQ Physical Limitation Score | 66.90 (26.09) | 64.47 (22.33) | 63.98 (26.77) | 68.41 (24.47) |
| Missing | 0 | 1 | 0 | 0 |
| KCCQ Quality of Life Score | 65.92 (25.19) | 62.50 (22.57) | 58.06 (24.84) | 63.69 (26.18) |
| KCCQ Social Limitation Score | 64.27 (28.65) | 64.44 (27.19) | 63.08 (29.00) | 64.81 (28.19) |
| Missing | 3 | 3 | 1 | 2 |
| 6-minute walk distance, meters | 324.43 (99.00) | 285.85 (93.40) | 294.90 (107.31) | 284.67 (112.12) |
| Missing | 0 | 0 | 0 | 1 |
| Weight (kilograms) | 100.05 (23.46) | 96.64 (21.18) | 97.02 (25.84) | - 1. 23.43) |

BMI indicates body mass index; eGFR, estimated glomerular filtration rate; KCCQ, Kansas City Cardiomyopathy Questionnaire; NYHA, New York Heart Association; NT-proBNP, N-terminal pro B-type natriuretic peptide. Values are shown as absolute numbers (percentages and mean ± SD or median with IQR).

^a^ An increase in ApoM was defined as a change greater than the median change observed (>0.00159) and for log NT-pro BNP, an increase was defined as a change greater than the median change observed (>0.692). Patients were categorized into four groups based on this: Decrease-Decrease, Decrease-Increase, Increase-Decrease, Increase-Increase.

^b^ Blood pressure and heart rate measured from noninvasive cuff measurements for patients in sinus rhythm and manual pulse and blood pressure for patients in atrial fibrillation
